# The BTPI: An online battery for measuring susceptibility to visual illusions

**DOI:** 10.1167/jov.23.10.2

**Published:** 2023-09-05

**Authors:** Yarden Mazuz, Yoav Kessler, Tzvi Ganel

**Affiliations:** 1 Department of Psychology and School of Brain Sciences and Cognition, Ben-Gurion University of the Negev, Beer-Sheva, Israel; 2 Department of Psychology and School of Brain Sciences and Cognition, Ben-Gurion University of the Negev, Beer-Sheva, Israel; 3 Department of Psychology and School of Brain Sciences and Cognition, Ben-Gurion University of the Negev, Beer-Sheva, Israel

**Keywords:** visual perception, visual illusions, individual differences, size perception

## Abstract

Visual illusions provide a powerful tool for probing the mechanisms that underlie perception. While most previous studies of visual illusions focused on average group-level performance, less attention has been devoted to individual differences in susceptibility to illusions. Unlike in other perceptual domains, in which there are established, validated tools to measure individual differences, such tools are not yet available in the domain of visual illusions. Here, we describe the development and validation of the BTPI (Ben-Gurion University Test for Perceptual Illusions), a new online battery designed to measure susceptibility to the influence of three prominent size illusions: the Ebbinghaus, the Ponzo, and the height–width illusions. The BTPI also measures perceptual resolution, reflected by the just noticeable difference (JND), to detect size differences in the context of each illusion. In Experiment 1 (*N* = 143), we examined performance in typical self-paced tasks, whereas in Experiment 2 (*N* = 69), we employed a fixed presentation duration paradigm. High test–retest reliability scores were found for all illusions, with little evidence for intercorrelations between different illusions. In addition, lower perceptual resolution (larger JND) was associated with a larger susceptibility to the illusory effect. The computerized task battery and analysis codes are freely available online.

## Introduction

Illusions provide an extreme case of a noticeable gap between the physical stimulus and its perceptual representation. In the visual domain, extensive research has been devoted to studying the perceptual and neural mechanisms that mediate illusions of size. Findings from behavioral and imaging studies demonstrate group differences in the susceptibility to visual illusions. In particular, it has been reported that illusions have different effects in different groups, such as in schizophrenia (King, Hodgekins, Chouinard, Chouinard, & Sperandio, 2017, but see Grzeczkowski, Clarke, Francis, Mast, & Herzog, 2017) and autism (Mitchell & Ropar, 2004, but see Hadad & Yashar, 2022), compared to controls. Moreover, previous research indicates that illusions have different effects in different cultures (Jahoda & Stacey, 1970) and different age groups (Cretenoud, Francis, & Herzog, 2020; Farquhart & Leibowitz, 1971; Grzeczkowski et al., 2017), as well as between different animal species (Santacà, Agrillo, & Miletto Petrazzini, 2021).

The observed group differences in susceptibility to illusions are also reflected in individual differences. Although relatively few studies have looked at individual differences in the susceptibility to visual illusions, findings indicate the existence of correlations between illusions' magnitude and factors such as visual acuity (Straßer, Kurtenbach, Langrová, Kuehlewein, & Zrenner, 2020), local–global processing style (Milne & Szczerbinski, 2009), spatial processing abilities (Axelrod, Schwarzkopf, Gilaie-Dotan, & Rees, 2017; Coren & Porac, 1987), and cognitive control (Shoshina & Shelepin, 2014). Imaging studies found that individual differences in the susceptibility to different illusions are associated with the amount of gray matter in distinct brain regions (Axelrod et al., 2017) and in the V1 surface (Schwarzkopf, Song, & Rees, 2011).

Grzeczkowski et al. (2017) tested correlations among different illusions to explore the possibility of a common factor governing susceptibility to illusions. While test–retest correlations showed stability (reliability) along with participants’ performance within each of the illusions, the between-illusions analysis, which consisted of 15 different comparisons, showed no significant correlations between illusions, with the only exception of a low correlation between the Ponzo and the Ebbinghaus illusions, both being visual illusions of size. Consistent with other studies in this domain, these findings indicate that different illusions are governed by independent, specific mechanisms (Coren et al., 1976; Cretenoud et al., 2019; Cretenoud, Francis et al., 2020; Cretenoud, Grzeczkowski, Bertamini, & Herzog, 2020).

Most studies that investigated visual illusions in general, and in particular individual differences, focused on the magnitude of the illusion by computing the point of subjective equality (PSE) of size perception in the context of the illusion (Axelrod et al., 2017; Bosten & Mollon, 2010; Coren et al., 1976; Coren & Porac, 1987; Cretenoud et al., 2019; Cretenoud, Francis et al., 2020; Cretenoud, Grzeczkowski et al., 2020; Farquhart & Leibowitz, 1971; Grzeczkowski et al., 2017; Jahoda & Stacey, 1970; Schwarzkopf et al., 2011; Shoshina & Shelepin, 2014). The PSE refers to the susceptibility to the illusion, also termed the *illusion's magnitude*; higher PSEs represent stronger susceptibility to the illusion. However, most previous studies did not consider a relevant potential psychophysical aspect of perception, the just noticeable difference (JND). The JND is defined as the minimum amount of stimulus magnitude (e.g., size) added to the stimulus in order to detect a difference. Therefore, lower JNDs indicate increased sensitivity to detect the slightest difference between stimuli of different sizes.

**Figure 1.**
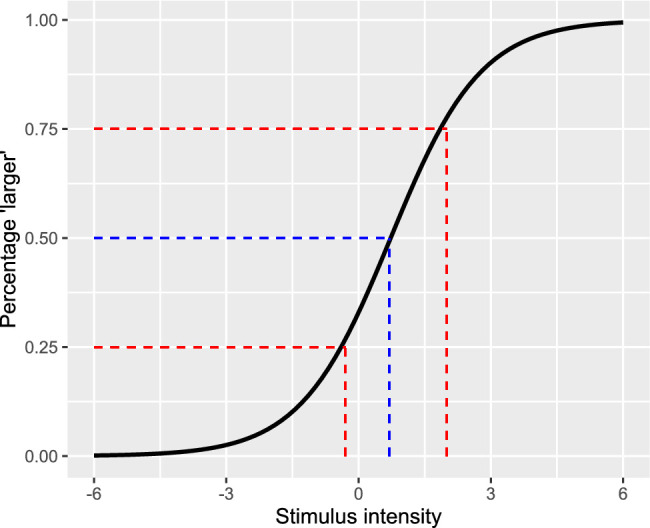
An illustration of the psychophysical curve. The x-axis represents the magnitude of the reference stimulus. The y-axis represents the percentage of trials by which the participant reported that the reference stimulus is larger than the standard stimulus, both embedded in the illusion. The black curve is the fitted sigmoid function that represents the participant's data. The blue line marks the PSE, the value in which the participant perceived both stimuli as equal. The red lines represent the area of uncertainty, which equals two JNDs.

The classic psychophysical method of constant stimuli allows computing the magnitude of the illusion based on the psychophysical function, which describes the relation between the relative sizes of a reference stimulus compared to a target stimulus, both embedded in the illusion. The point at which the two stimuli are perceived as equal (the PSE) represents the magnitude of the illusion. The slope of the fitted function represents the JND, the sensitivity to detect differences in size between the two stimuli (Zitron-Emanuel & Ganel, 2018; for illustration, see Figure 1). Here, we took advantage of this method, modified for the purposes of the current study, to provide a comprehensive measure of individual differences in terms of both the susceptibility to visual illusion and the perceptual resolution to size.

There is a large and growing body of standardized measurements for individual differences among different domains of cognitive performance. Examples include the Raven's Progressive Matrices to measure intelligence (Raven, 2000), complex span tasks for the measurement of working memory capacity (Redick et al., 2012), and the Cambridge Face Memory Test for face recognition (Duchaine & Nakayama, 2006; Murray & Bate, 2020). At present, however, there is no standardized tool to measure performance and individual differences along the susceptibility to illusions. This lack of a standard tool can lead to increased variance between experimental designs, measurement methods, and statistical analyses that could account for the mixed pattern of results in measures of susceptibility for different illusions (Anderson, Tan, & Marlow, 2019; Santacà et al., 2021; Skottun & Skoyles, 2014).

**Figure 2.**
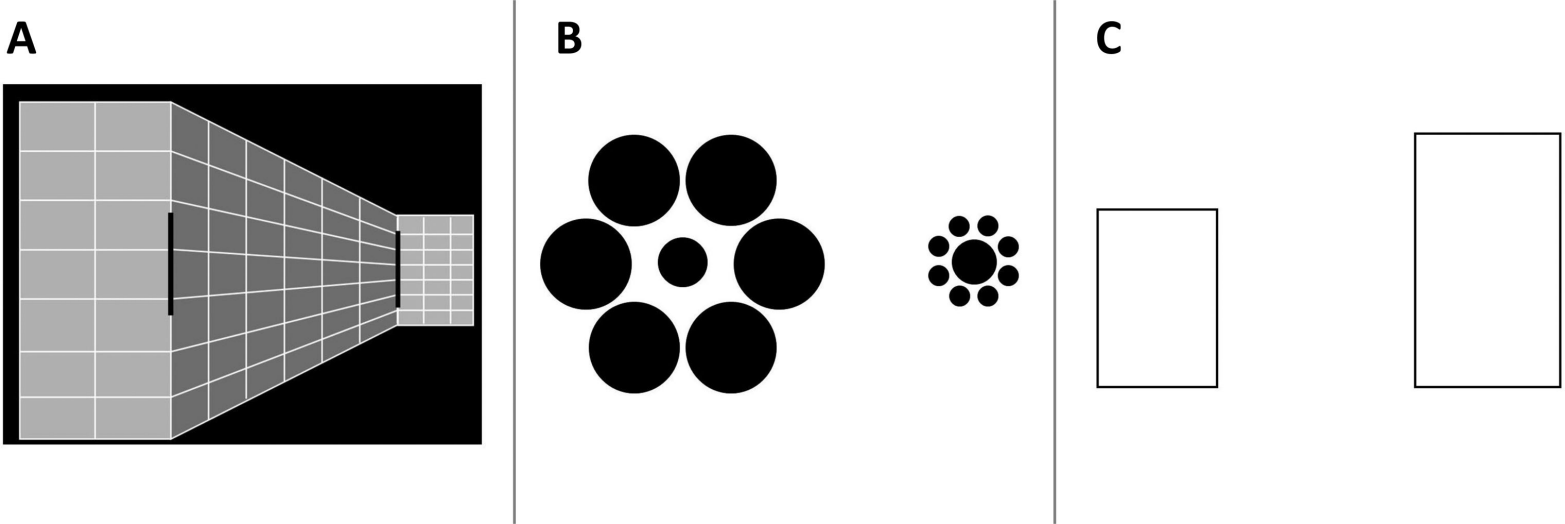
The stimuli used in Experiments 1 and 2. (**A**) Ponzo illusion—the participants were instructed to choose the *longer* object. (**B**) Ebbinghaus illusion—the participants were instructed to choose the larger *central* circle. (**C**) Height–width illusion—the participants were instructed to choose the *wider* rectangle.

Here, we focused on illusions in the visual domain to further understand the different visual mechanisms that govern each illusion. For example, the mechanism that underlies the Ponzo illusion (see Figure 2A) has been attributed to pictorial depth perception, particularly pictorial cues that establish size constancy in everyday situations. The Ponzo illusion provides pictorial distance cues such as perspective lines that activate top-down mechanisms of size constancy, in which some objects appear to be located further away from the observer. That makes people perceive these objects as larger than objects that appear closer, even in cases where their retinal representation is equal (Sperandio & Chouinard, 2015).

The primary purpose of the present study is to build and validate a standardized battery for measuring individual differences in perception of size illusions—the BTPI (Ben-Gurion University Test for Perceptual Illusions). Currently, our main focus is on visual size illusions, but this tool could be extended in principle to other illusions in different domains. The BTPI will assess (a) the degree of susceptibility to three different size illusions (by computing the PSE) and (b) the resolution to size differences in the context of each illusion (the JND). We focused on three major visual size illusions: the Ponzo illusion (Ganel, Tanzer, & Goodale, 2008; Leibowitz, Brislin, Perlmutrer, & Hennessy, 1969; Ozana & Ganel, 2020), the Ebbinghaus illusion (Coren & Miller, 1974), and the height–width illusion (Ben-Shalom & Ganel, 2012; Ganel & Goodale, 2003; Zitron-Emanuel et al., 2022; Zitron-Emanuel & Ganel, 2020). Each of the illusions taps a different aspect of size-related visual processing. The mechanism of size constancy may trigger the Ponzo illusion, the Ebbinghaus illusion is mediated by size-contrast effects, and the height–width illusion reflects the holistic processing of object shape. Individual differences along this set of visual illusions are measured using the same unified tool, which is accessible online to the research community. In Experiment 1, the stimuli for each illusion were presented in a typical self-paced manner. In Experiment 2, we employed a fixed presentation duration paradigm (1,000 ms) in order to control for possible effects of presentation duration that could affect performance for some illusions (Bressan & Kramer, 2021). Based on the combined results of the two experiments, we offer a unified tool to measure the susceptibility and the visual resolution for size for each of the three illusions. The BTPI is freely accessible online to the research community.

## Experiment 1

### Method

#### Participants

For the initial session, 204 participants (102 males) were recruited via the Prolific website. The session’s duration was approximately 20 min, and the participants received 3.13 euros for their participation. Only participants with a goodness of fit (GOF; see Method below) higher than 0.4 in each of the illusions were invited to participate in the second session. For the overall analysis, data for illusions with GOF smaller than 0.7 were excluded from further analysis. Overall, the data of 143 participants (73 males, *M*_age_ = 26.8, *SD*_age_ = 8.9, age range = 18–61), who completed the two sessions, were analyzed in the experiment. As shown in Table 1, 119 out of the total 143 participants passed the GOF criterion for the Ponzo illusion sessions, 133 out of 143 participants passed the criterion for the Ebbinghaus illusion, and 136 participants passed the criterion for the height–width illusion. All participants signed a consent form prior to beginning the experiment. The experiments were approved by the BGU (Ben-Gurion University of the Negev) ethics committee.

**Table 1.** Model comparison. Model 1 = two-parameter model; Model 2 = three-parameter model.

	Ponzo	Ebbinghaus	Height–width
GOF, Model 1	0.83	0.88	0.9
GOF, Model 2	**0.85**	0.88	**0.92**
Log-likelihood, Model 1	13.232 (*df* = 3)	17.037 (*df* = 3)	24.923 (*df* = 3)
Log-likelihood, Model 2	13.242 (*df* = 4)	17.037 (*df* = 4)	24.923 (*df* = 4)
BIC, Model 1	−**19.01**	−**26.619**	−**42.391**
BIC, Model 2	−16.545	−24.134	−39.91

The bold values indicate the preferred model.

#### Stimuli

The susceptibilities to the Ponzo, the Ebbinghaus, and the height–width illusions were tested in three different experimental blocks in each experimental session. For all illusions, one object served as the standard stimulus and was fixed in size. Twelve reference stimuli, smaller or bigger than the standard stimulus in fixed intervals that differed for each illusion, were presented alongside with the standard stimulus in the context of the illusion. The sizes of the standard and reference stimuli for each of the illusions were calculated based on pilot experiments designed to identify the ideal range required to detect differences along the susceptibility to each illusion. The left–right location of the standard and reference stimulus on the screen was counterbalanced for each participant.

The background of the Ponzo illusion consisted of perspective lines and other pictorial depth cues designed to make one side of the display appear to be further away in depth than the other side (Figure 2A). Two rectangular objects were presented on the two sides of the illusory background. We note that the version of the Ponzo illusion used in the current design is different from the classic version of the illusion, in that it contains other depth cues beyond two-dimensional (2D) perspective lines. Yet, given that the additional cues provided here are 2D cues that also indicate depth, and based on previous studies in which we and others refer to this enhanced version of the illusion as the Ponzo illusion (e.g., Ben-Shalom & Ganel, 2012; Ganel et al., 2008; Gonzalez, Ganel, & Goodale, 2006, Gonzalez, Ganel, Whitwell, & Goodale, 2008; Navon & Ganel, 2020; Whitwell, Buckingham, Enns, Chouinard, & Goodale, 2016), we prefer to keep a similar terminology in the present design. The size of the standard stimulus was 151 pixels, and it was always presented on the “further away” side of the illusion. One of the 12 reference stimuli was presented on the opposite side of the illusion. The smallest reference stimulus was scaled to be 5% larger than the standard, and the other 11 reference stimuli were scaled larger in constant intervals of 7%. The display of the Ebbinghaus illusion contains two circles located side by side (Figure 2B). Eight smaller circles surrounded the standard circle (85 pixels), and six bigger circles surrounded the reference circle (starting from 110% of the standard, with fixed intervals of 5%). In the height–width illusion, the stimuli were two rectangles presented side by side (Figure 2C). Their heights were fixed (453 and 317 pixels). The width of the taller rectangle, which served as the standard, was 260 pixels. We created 12 different widths for the short rectangle, ranging between 82% and 118% in width compared to the width of the standard rectangle, with fixed intervals of 3% between the stimuli.

#### Design and procedure

Our first objective was to validate the tool by measuring its test–retest reliability. Therefore, the experiment included two identical sessions that occurred on 2 consecutive days.

An adapted version of the method of constant stimuli was used for each illusion. Following the instructions, there were six training trials to familiarize the participants with the task. Each trial began with a fixation cross presented for 1,000 ms, followed by the presentation of the target stimuli for 3,000 ms. Each illusion was presented in a separate block with a fixed block order of Ponzo, Ebbinghaus, and then the height–width illusion. Within each block, stimuli were presented in a randomized fashion (12 repetitions of each of the 12 standard-reference combinations, 144 trials overall). For each standard-reference combination, the standard stimulus was presented an equal number of times on the right and left sides of the display. Participants were asked to choose the longer stimulus for the Ponzo illusion, the bigger circle for the Ebbinghaus illusion, and the wider rectangle for the height–width illusion. The answer was indicated by keyboard response (K, the object on the right; S, the object on the left). The subsequent trial was displayed after the participant's response or 3,000 ms after the presentation of the stimulus. After 3,000 ms, the next trial was automatically initiated.

#### Data analysis

Trials in which the participant did not answer within time limit (3,000 ms) were excluded from the analysis. For each participant, we calculated the proportion of trials in which the participant reported that the reference stimulus was larger (or wider) than the standard. We then fitted two models to the data. The first two-parameter model was the sigmoid function $\frac{1}{1+e^{-\frac{x-A}{B}}}$. This model assumes that performance in the task solely relies on perceptual judgments. The second model included another “lapse rate” parameter. Following the logic of Wichmann and Hill (2001), we applied a mixture model in which the lapse rate was added to the model as a free parameter. Addended trials followed a psychophysical function, whereas the responses in lapsed trials were determined by a “coin flip”—namely, randomly choosing between the left/right responses with equal probability. The mixture parameter *p*—namely, the probability of lapse responses—was estimated as part of the model. Model comparison was based on the BIC (Bayesian information criterion) statistic, which penalizes for the number of parameters (BIC = –2 * log-likelihood + log(*N*)*K, where *N* is the number of observations and *K* is the number of parameters). Based on the sigmoid function of each model, we extracted the values of PSE, constant error (CE), JND, and GOF, being the squared correlation between observed and fitted values. The CE represents the magnitude of the illusion and was computed by subtracting the value of the PSE (50% “larger” responses) from the value of the standard stimulus. The JND represents a perceptual resolution to size differences in the context of the illusion and was calculated by dividing the range between 25% and 75% of the function by 2 (Figure 1). For clarity, we transformed the CE and the JND raw scores to percentage scores for each participant, representing the magnitude of the illusion, and the magnitude of the JND in percentages compared to the standard stimulus. Reaction times (RTs) were also measured in each trial, and the mean RT was calculated for each participant and in each illusion.

A test–retest reliability was assessed by the correlation between the two sessions of each illusion. In addition, we calculated the correlations between the average CEs and JNDs in each illusion. To examine if the exposure time to the illusions affected the susceptibility to the illusion, we calculated the correlation between RTs (which also corresponds to exposure time in Experiment 1) and between CEs for each task. Comparisons between the reliabilities of the CEs and JNDs of the illusions for nonoverlapping groups were performed using the “cocor” package in R (Diedenhofen & Musch, 2015).

### Results and discussion

#### Model comparison

The two models were fitted to the data across both sessions. The lapse rate mean values in Model 1 were 0.064 (*SD* = 0.141) for the Ponzo task, 0.053 (*SD* = 0.141) for the Ebbinghaus task, and 0.062 (*SD* = 0.130) for the height–width task. Table 1 presents the model comparison statistics. The BIC statistic favored the more parsimonious Model 1 in all the illusions. Accordingly, the following analyses were carried out using this model.

#### Main analysis

Table 2 displays the average CEs and JNDs in each illusion divided by session. The mean values of the CE and JND for the Ponzo illusion were 44.81% (*SD* = 20.97) and 10.71% (*SD* = 4.68), respectively. The average magnitude of the Ebbinghaus illusion (CE) was 44.32% (*SD* = 13.33), and the mean JND was 7.5% (*SD* = 3.28). The CE for the height–width illusion was 2.89% (*SD* = 3.51), and the JND was 3.97% (*SD* = 1.66).

**Table 2.** The CEs and JNDs for each illusion (in percentages), Experiment 1. The CE represents the magnitude of the illusion. The JND represents visual resolution for difference in size.

Illusion	Session	*N*	Measure	Mean	*SD*	Skewness	Kurtosis
Ponzo	1	125	CE	47.82	24.85	0.67	0.09
			JND	12.47	7.06	1.48	2.94
	2	119	CE	45.09	23.47	0.59	0.66
			JND	9.81	4.63	1.01	1.23
Ebbinghaus	1	131	CE	45.53	14.69	0.26	−0.23
			JND	8.06	4.11	1.19	2.08
	2	133	CE	45.26	14.44	0.29	−0.34
			JND	7.21	3.86	1.87	5.91
Height–width	1	138	CE	2.55	5.38	−3.44	21.85
			JND	4.41	2.31	1.32	2.94
	2	136	CE	3.12	3.43	−0.97	2.84
			JND	3.76	1.84	1.02	1.97

#### Reliability

**Figure 3.**
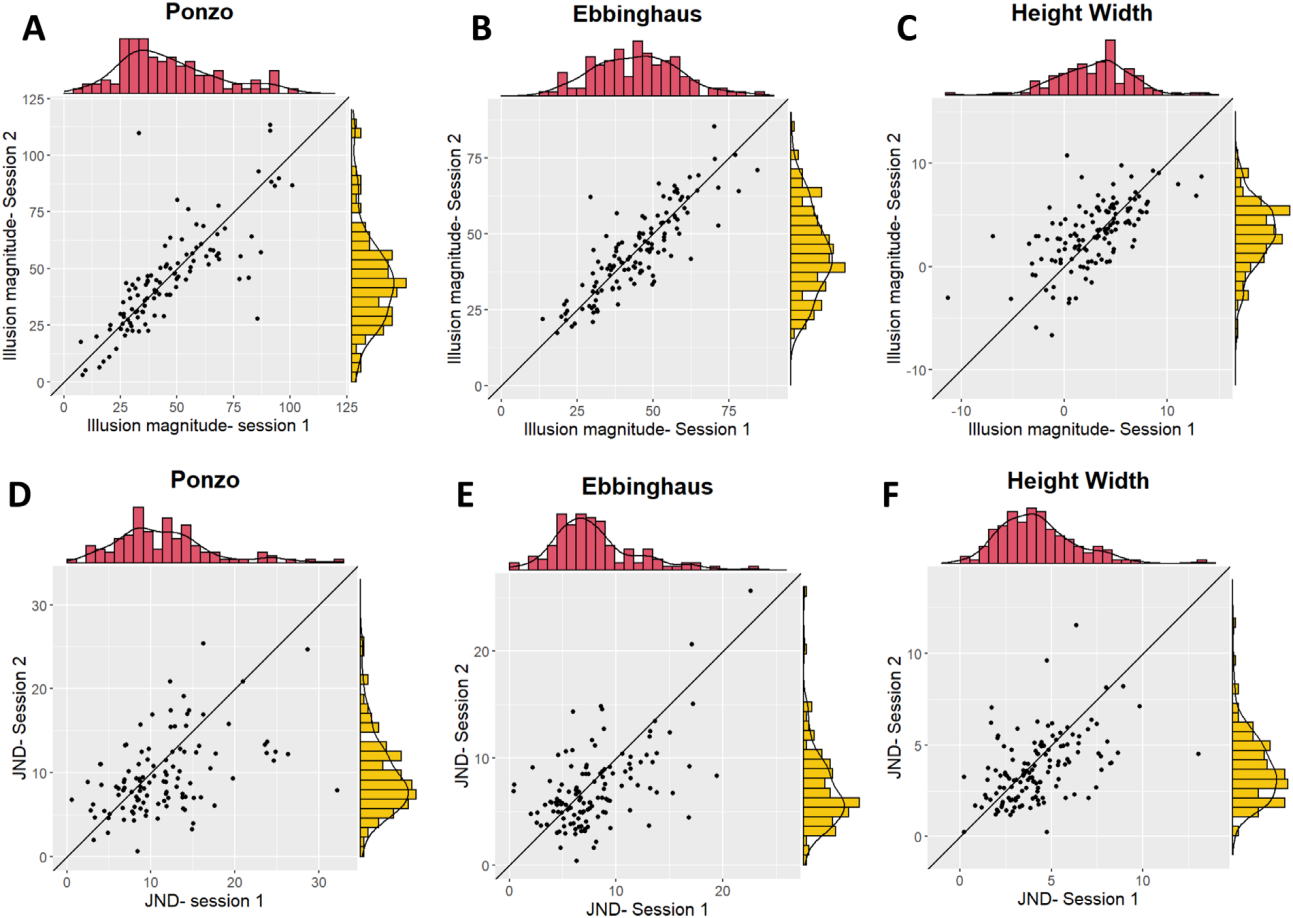
Test–retest reliabilities for the illusion magnitudes and JNDs in Experiment 1. Each dot on the scatterplot represents one participant. The top panel (**A**, Ponzo; **B**, Ebbinghaus; **C**, height–width) shows the correlations for the illusion magnitude, and the bottom panel (**D**, Ponzo; **E**, Ebbinghaus; **F**, height–width) shows the correlations for the JNDs. The distributions of the two sessions are presented in the red and yellow histograms.

Pearson correlations were computed to assess the test–retest reliabilities. As shown in Figure 3, there were high test–retest correlations between the magnitudes of the illusions in the two sessions (Ponzo: *r*(109) = .80, *p* < 0.001; Ebbinghaus: *r*(122) = .85, *p* < 0.001; height–width: *r*(131) = 0.70, *p* < 0.001). The test–retest correlations for the JNDs in the two sessions were *r*(109) = .50, *p* < 0.001 for the Ponzo illusion, *r*(122) = .54, *p* < 0.001 for the Ebbinghaus illusion, and *r*(131) = .47, *p* < 0.001 for the height–width illusion (see Figure 3). The differences between the reliabilities of the CE and JND within each illusion were significant (Ponzo: *z* = 4.68, *p* < 0.001; Ebbinghaus: *z* = 5.72, *p* < 0.001; height–width: *z* = 3.18, *p* = 0.001).

The relatively lower reliability scores of the JNDs may be related to the fact that the tasks were mainly targeted to detect a broad range of individual differences along the CEs of the illusions in expanse for a relatively large gap differences between the predefined intervals of the reference stimulus used in our design. We will discuss this issue further in the General discussion.

#### Correlations between measures

As illustrated in the correlations table (Table 3), there was a positive correlation between the magnitudes of the Ponzo and Ebbinghaus illusions. Similarly, there was a smaller, yet significant positive correlation between the magnitudes of the Ponzo and height–width illusions. These results indicate that individuals with a higher susceptibility to the Ponzo illusion would tend to show higher susceptibilities to the Ebbinghaus and height–width illusions.

**Table 3.** Means, standard deviations, and Pearson correlations between the different measures of each illusion (across sessions) in Experiment 1. CE and JND values are in percentages. RT values are in ms. Values in square brackets indicate 95% confidence intervals. *p < 0.05. **p < 0.01.

Variable	*M*	*SD*	1	2	3	4	5	6	7	8
1. CE Ponzo	44.81	20.97								
2. JND Ponzo	10.71	4.68	.37**							
			[.20, .52]							
3. RT Ponzo	980.02	230.30	.12	−.01						
			[−.07, .30]	[−.19, .18]						
4. CE Ebbinghaus	44.37	13.33	.31**	.18	−.22*					
			[.12, .48]	[−.02, .36]	[−.40, −.03]					
5. JND Ebbinghaus	7.50	3.28	.05	.28**	−.28**	.43**				
			[−.14, .25]	[.09, .45]	[−.46, −.09]	[.28, .56]				
6. RT Ebbinghaus	964.28	229.25	.10	−.01	.76**	−.41**	−.29**			
			[−.09, .29]	[−.21, .19]	[.66, .83]	[−.55, −.25]	[−.44, −.12]			
7. CE height–width	2.89	3.51	.20*	.05	−.23*	.07	.03	−.19*		
			[.01, .38]	[−.14, .24]	[−.40, −.04]	[−.11, .24]	[−.15, .20]	[−.36, −.02]		
8. JND height–width	3.97	1.66	−.00	.22*	−.34**	.18*	.45**	−.27**	−.12	
			[−.20, .19]	[.03, .40]	[−.50, −.15]	[.01, .35]	[.30, .58]	[−.42, −.09]	[−.29, .05]	
9. RT height–width	935.84	211.94	.09	.03	.82**	−.22*	−.20*	.84**	−.30**	−.16
			[−.11, .27]	[−.16, .22]	[.75, .87]	[−.38, −.04]	[−.36, −.02]	[.77, .88]	[−.45, −.13]	[−.32, .01]

Pearson correlations were computed to assess the test–retest reliabilities. As shown in Figure 3, there were high test–retest correlations between the magnitudes of the illusions in the two sessions (Ponzo: *r*(109) = .80, *p* < 0.001; Ebbinghaus: *r*(122) = .85, *p* < 0.001; height–width: *r*(131) = 0.70, *p* < 0.001). In addition, we computed Pearson correlations between the JNDs in the two sessions. The test–retest correlations for the JNDs in the two sessions were *r*(109) = .50, *p* < 0.001 for the Ponzo illusion; *r*(122) = .54, *p* < 0.001 for the Ebbinghaus illusion; and *r*(131) = .47, *p* < 0.001 for the height–width illusion (see Figure 3).

A positive correlation between the CEs and JNDs of the Ponzo and the Ebbinghaus illusions indicated that subjects with a lower perceptual resolution for size differences showed increased susceptibility to the illusions. Unlike the Ponzo and Ebbinghaus illusions, there was no correlation between the CE and the JND for the height–width illusion (Table 3).

**Figure 4.**
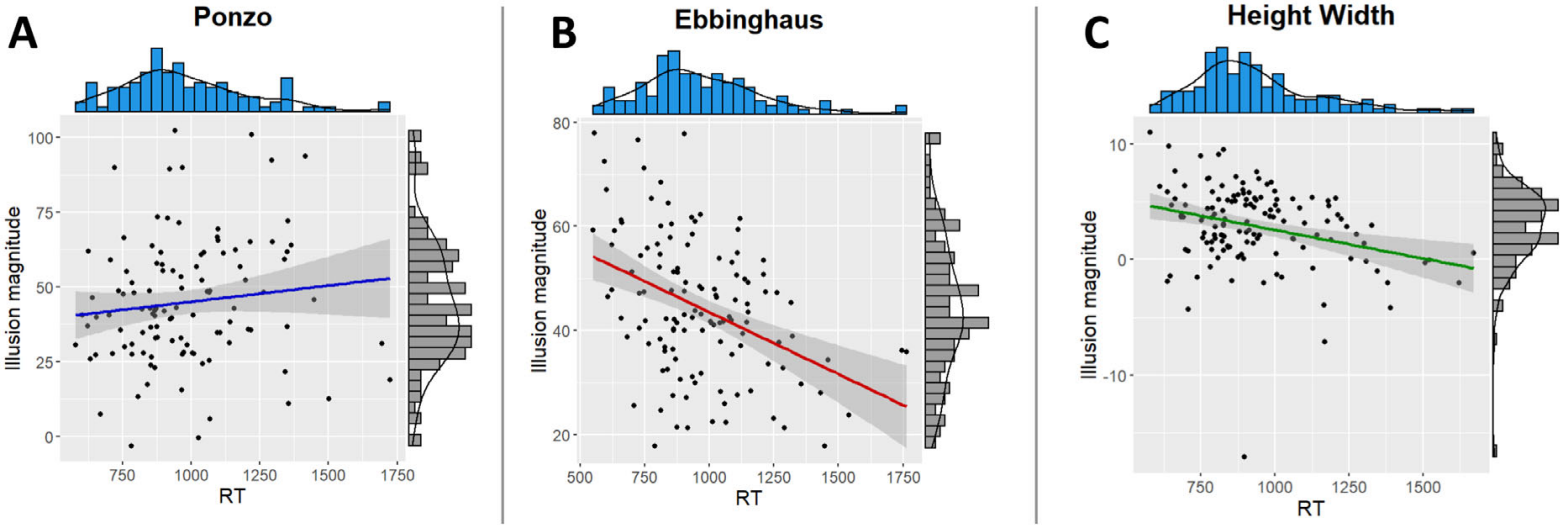
Correlation between RT (which equals exposure time in Experiment 1) and the illusion magnitude in Experiment 1. The solid lines in each graph represent the linear regression (**A**, Ponzo; **B**, Ebbinghaus; **C**, height–width).

Recently, Bressan and Kramer (2021) suggested that, at least in the case of the Ebbinghaus illusion, exposure time to the illusory display is confounded with the illusion's magnitude. These authors showed that larger exposure times resulted in a smaller susceptibility to the illusion. We therefore explored the correlation between the illusion magnitude and exposure time for all three illusions. We remind the reader that in the current design, the RT equals the exposure time of the illusion. We, therefore, computed the correlation between illusions’ magnitudes and RT. As can be seen in Table 3, there was a negative correlation between the magnitude of the illusion and RT for the Ebbinghaus and height–width illusions, indicating that individuals with longer exposure times had a lower susceptibility to these illusions (Figure 4). Unlike these two illusions, there was no effect of exposure duration on the susceptibility to the Ponzo illusion.

Given that exposure duration could mediate the amount of susceptibility to two out of the three illusions tested in the BTPI, we modified the design of Experiment 1 so that exposure times would not be a confounding factor for the magnitude of the illusion. In Experiment 2, we used a similar design to the one used in Experiment 1, with fixed exposure times of 1,000 ms.

## Experiment 2

To eliminate the possible confound of exposure duration on the illusions’ magnitudes, the design of Experiment 2 was similar in all aspects to the one used in Experiment 1 but with a fixed exposure duration of 1,000 ms.

### Method

#### Participants

In total, 125 participants (60 males) were recruited via the Prolific website for the initial session, which lasted approximately 20 min, and received 3.13 euros for their participation. We used the same inclusion criteria as in Experiment 1. A total of 69 participants (35 males) were included in the analysis (*M*_age_ = 39.2, *SD*_age_ = 11.9, age range = 21–60). Prior to the experiment, each participant signed a consent form, agreeing to participate in the experiment.

#### Stimuli, design, and procedure

The stimuli were identical to the stimuli in used Experiment 1. As in Experiment 1, Experiment 2 included two identical sessions that occurred on consecutive days. The procedure was similar to the one used in Experiment 1 in all aspects but one. Trials began with a fixation cross that was presented for 1,000 ms, followed by the presentation of the stimuli for 1,000 ms, after which the stimuli disappeared, and the participant was asked to type their response (Bressan & Kramer, 2021). Participants could not respond during the 1,000-ms presentation duration of the stimuli. Again, during the response period, participants were asked to indicate which object was larger, bigger, or wider (Ponzo, Ebbinghaus, and height–width, respectively). The subsequent trial was initiated if there was no response after 3,000 ms.

#### Data analysis

JNDs, CEs, and GOFs were calculated as in Experiment 1. As detailed earlier, in Experiment 1, the RT count initiated with the presentation of the stimuli and ended with the participant's response. In the current experiment, RT count was initiated when the participants were presented with the response display (following exposure to the stimuli) and ended with their responses; hence, RT measures in Experiment 2 did not include exposure durations to the illusion.

### Results and discussion

#### Model comparison

The two models were fitted to the data across both sessions. The lapse rate mean values in Model 2 were 0.063 (*SD* = 0.124), 0.039 (*SD* = 0.095), and 0.048 (*SD* = 0.113) for the Ponzo, Ebbinghaus, and height–width tasks, respectively. Table 4 presents the model comparison statistics. The BIC statistic favored the more parsimonious Model 1 in the Ponzo and height–width illusions, but not in the Ebbinghaus illusion data. Despite the latter finding, and taking the results of Experiment 1 into consideration, we decided to model the data using Model 1 for all the illusions.

**Table 4.** Model comparison. Model 1 = two-parameter model; Model 2 = three-parameter model.

	Ponzo	Ebbinghaus	Height–width
GOF, Model 1	0.86	0.88	0.93
GOF, Model 2	**0.88**	**0.89**	**0.94**
Log likelihood, Model 1	21.212 (*df* = 3)	5.387 (*df* = 3)	11.213 (*df* = 3)
Log likelihood, Model 2	21.170 (*df* = 4)	8.270 (*df* = 4)	11.213 (*df* = 4)
BIC, Model 1	−**34.969**	−3.32	−**14.972**
BIC, Model 2	−32.34	−**6.6**	−12.487

The bold values indicate the preferred model.

The descriptive statistics of the illusions’ magnitudes and JNDs in both sessions are presented in Table 5. The mean magnitudes of the CE and JND for the Ponzo illusion were 46.70% (*SD* = 20.90) and 10.70% (*SD* = 4.81), respectively. The magnitude of the Ebbinghaus illusion (i.e., CE) was 40.50% (*SD* = 2.98), and the JND was 6.74% (*SD* = 3.02). The magnitude of the height–width illusion was 4.38% (*SD* = 3.15), and the JND was 3.76% (*SD* = 1.48).

**Table 5.** The CEs and JNDs for each illusion (in percentages), Experiment 2. The CE represents the magnitude of the illusion. The JND represents visual resolution for difference in size.

Illusion	Session	*N*	Measure	Mean	*SD*	Skewness	Kurtosis
Ponzo	1	66	CE	46.16	23.31	0.54	0.41
			JND	12.70	6.79	0.74	0.28
	2	63	CE	47.37	23.55	0.55	0.50
			JND	9.10	5.13	1.23	1.39
Ebbinghaus	1	68	CE	40.87	3.39	0.42	−0.21
			JND	7.49	4.07	2.46	9.88
	2	65	CE	40.19	2.91	0.18	−0.54
			JND	6.01	2.99	0.77	0.47
Height–width	1	66	CE	4.31	3.48	6.34e-03	−0.04
			JND	4.30	2.04	1.28	1.06
	2	68	CE	4.18	3.57	−0.54	0.82
			JND	3.25	1.42	0.34	1.41

#### Reliability

**Figure 5.**
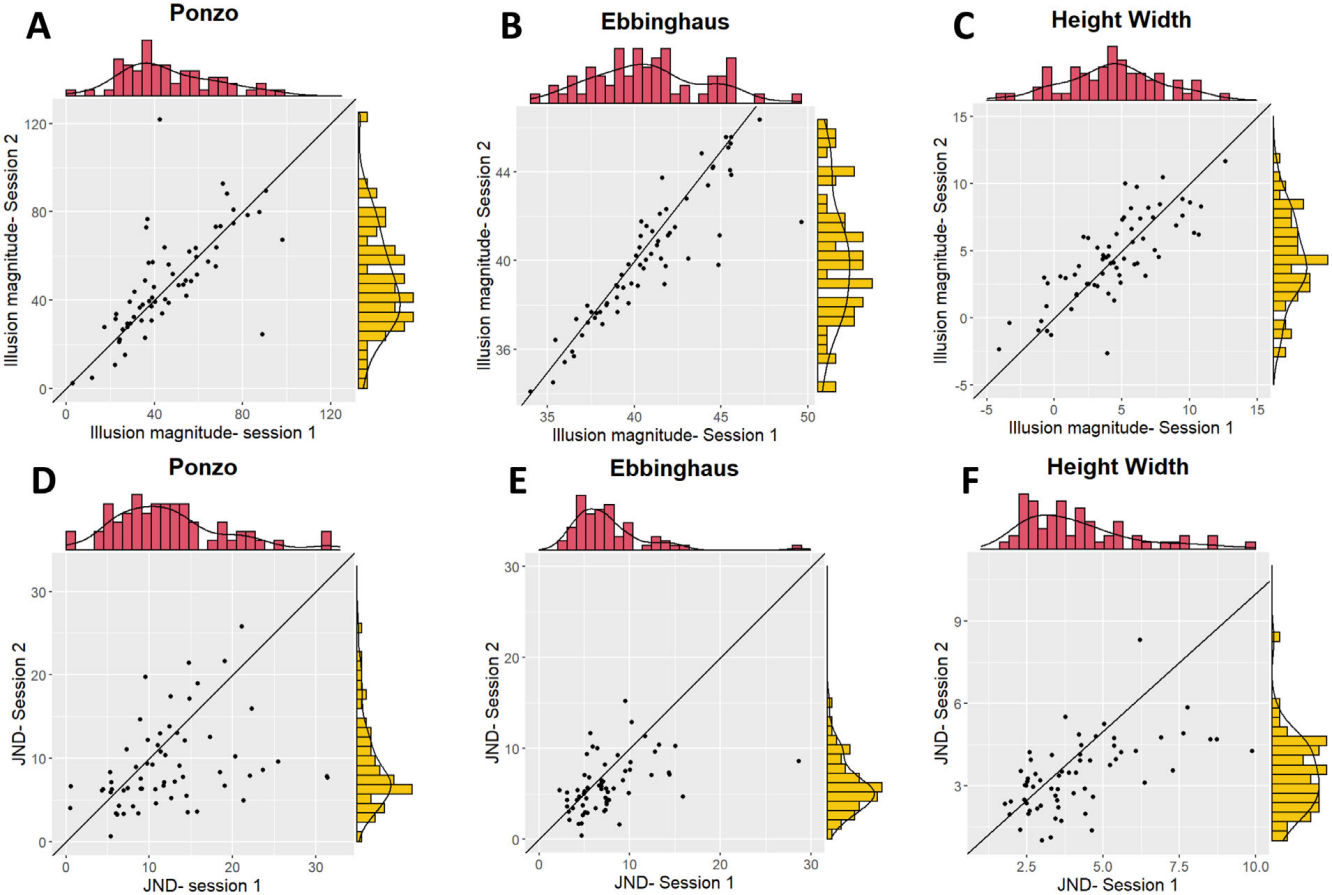
Test–retest reliabilities for the illusion magnitudes and JNDs in Experiment 2. Each dot on the scatterplot represents one participant. The top panel (**A**, Ponzo; **B**, Ebbinghaus; **C**, height–width) shows the correlations for the illusion magnitude, and the bottom panel (**D**, Ponzo; **E**, Ebbinghaus; **F**, height–width) shows the correlations for the JNDs. The distributions of the two sessions are presented in the red and yellow histograms.

The test–retest reliabilities are presented in Figure 5. Notably, the Ponzo test–retest reliability in Experiment 2 was relatively low compared to the one found in Experiment 1 (*r*(60) *=* .71, *p* < 0.001), which suggests that limited exposure time is not ideal to study individual differences in the susceptibility to the Ponzo illusion. Unlike the Ponzo illusion, test–retest reliabilities for the Ebbinghaus and height–width illusions were even higher than the ones found in Experiment 1 (Ebbinghaus: *r*(62) = .89, *p* < 0.001; height–width: *r*(63) = .78, *p* < 0.001). These results are in agreement with the correlations found between exposure times and the illusions’ magnitudes in Experiment 1 and indicate that measuring individual differences along the susceptibility to these two illusions is more reliable when exposure time is fixed.

The test–retest reliability for the JND was *r*(60) = .32, *p* = 0.01 for the Ponzo illusion, *r*(62) = .44, *p* < 0.001 for the Ebbinghaus illusion, and *r*(63) = .57, *p* < 0.001 for the height–width illusion (Figure 5). Similarly to Experiment 1, the differences between the reliability of the CEs and JNDs of the three illusions were significant (Ponzo: *z* = 3.32, *p* = 0.001; Ebbinghaus: *z* = 6.01, *p* < 0.001; height–width: *z* = 2.35, *p* = 0.019).

#### Correlations between measures

The correlations between the different measures are presented in Table 6. As in Experiment 1, there were positive correlations between the CEs and the JNDs of the Ponzo and Ebbinghaus illusions. Unlike Experiment 1, there was an unpredicted, negative correlation between the CE and the JND in the height–width illusion. Unlike Experiment 1, the correlations between the Ponzo and Ebbinghaus illusions and between the Ponzo and height–width illusions were not significant. This may be due to the decrease in reliability of the Ponzo illusion observed in Experiment 2.

**Table 6.** Means, standard deviations, and Pearson correlations between the different measures of each illusion (across sessions) in Experiment 2. CE and JND values are in percentages. RT values are in ms. Values in square brackets indicate 95% confidence intervals. *p < 0.05. **p < 0.01.

Variable	*M*	*SD*	1	2	3	4	5	6	7	8
1. CE Ponzo	46.67	20.92								
2. JND Ponzo	10.73	4.81	.39**							
			[.15, .58]							
3. RT Ponzo	458.09	156.07	−.01	.13						
			[−.26, .24]	[−.12, .37]						
4. CE Ebbinghaus	40.46	2.98	.14	−.04	−.05					
			[−.12, .39]	[−.29, .23]	[−.31, .21]					
5. JND Ebbinghaus	6.74	3.02	.13	.03	−.16	.61**				
			[−.13, .38]	[−.23, .29]	[−.41, .10]	[.42, .74]				
6. RT Ebbinghaus	394.81	155.53	−.14	−.05	.68**	.05	−.04			
			[−.39, .12]	[−.31, .21]	[.51, .80]	[−.19, .30]	[−.29, .20]			
7. CE height–width	4.38	3.15	−.04	−.10	.05	.02	.04	.05		
			[−.29, .22]	[−.35, .16]	[−.20, .31]	[−.23, .27]	[−.21, .29]	[−.20, .30]		
8. JND height–width	3.76	1.48	.19	.23	−.05	.37**	.40**	.11	−.28*	
			[−.07, .42]	[−.03, .45]	[−.30, .21]	[.13, .57]	[.17, .59]	[−.15, .35]	[−.49, −.04]	
9. RT height–width	364.29	114.73	−.04	.02	.83**	.10	−.05	.81**	.03	.10
			[−.29, .22]	[−.24, .28]	[.73, .90]	[−.15, .34]	[−.30, .20]	[.71, .88]	[−.21, .27]	[−.14, .34]

### General discussion

The main purpose of the current study was to develop and validate an online tool that measures individual differences in susceptibility to visual illusions, focusing on visual illusions of size. A major advantage of our new tool is its ability to measure individual differences in sensitivity to visual size differences as an inherent part of the measurement. Importantly, in addition to the potential of the new tool to explore different venues in human perception of size and their interactions with many aspects of human performance, the current findings already suggest some theoretical insights about the mechanisms that govern the perception of size.

The test–retest results for the illusion magnitude were remarkedly high for almost all illusions. One exception was the reliability of the Ponzo illusion in Experiment 2, which was lower than the reliability found in Experiment 1. This finding indicates that in the case of the Ponzo illusion, the fixed, short exposure time limit does not provide ideal conditions to measure the illusion's magnitude. Given that the results of both experiments clearly show that unlike the other illusions, the Ponzo illusion is not confounded by exposure duration, we decided to use the initial, unrestricted version of the Ponzo illusion in the final version of the BTPI. In line with the same logic, the reliabilities for the Ebbinghaus and the height–width illusions were higher in Experiment 2, probably because exposure durations were fixed and did not interfere with the measurement of the illusions’ magnitude. Thus, our results reinforce the notion that some visual illusions are affected by exposure time (Bressan & Kramer, 2021) but also show that other illusions show no influence of exposure time and are even disrupted when fixed, short exposure time limits are used. The final version of the BTPI therefore includes the Ponzo task from Experiment 1 and the Ebbinghaus and height–width tasks from Experiment 2.

Previous findings have already implied stable individual differences along the within-domain susceptibility to visual illusions, reflected by high correlations between individual scores in a given illusion across time, measurement method, and different variants of the illusion (Coren & Porac, 1987; Cretenoud et al., 2019, Cretenoud, Grzeczkowski, Kunchulia, & Herzog, 2021; Cretenoud, Grzeczkowski et al., 2020; Grzeczkowski et al., 2017). Our findings reinforce this idea and provide a standardized tool to measure individual differences in the susceptibility to illusions.

We note that unlike the high reliability scores of the illusions’ magnitudes, reliability scores of the perceptual resolution to detect size differences were significantly lower. These lower scores may have resulted from the values of predefined intervals between the reference stimulus in our design. Our main purpose in developing the BTPI was to capture the largest range of individual differences along the susceptibility (CEs) to each illusion. For example, the size ratio between the smallest and the largest reference stimuli was 84% for the Ponzo illusion, 60% for the Ebbinghaus illusion, and 36% for the height–width illusion. Given that the number of different reference stimuli in the current design was constant (12), increasing the smallest/largest ratio would result in a similar increase in the gap between successive references, namely, in the resolution of the measurement. This could result in reduced sensitivity to capture small individual differences along JNDs. For example, for the Ponzo illusion, the large size range resulted in relatively large gaps of 7% between adjacent reference stimuli. Such a large gap could restrict the sensitivity of the BTPI in capturing small individual differences in JNDs (smaller than 7%). A similar argument could apply for the Ebbinghaus illusion (a range of 60%, 5% gaps). We note, however, that the relatively small gap between references (3%) in the height–width illusion could allow, in principle, larger sensitivity to measure JNDs. This is indicated by the differences between the reliabilities of the JNDs in Experiment 2. The reliability of the JND for the height–width illusion was relatively high (0.47) compared to the reliabilities of JNDs in the Ponzo (0.32) and Ebbinghaus (0.44) illusions in this experiment. In principle, we could have used an expanded design with a larger number of reference stimuli and, therefore, with smaller size gaps. However, such a design would increase the length of the experiment and restrict the potential number of illusions measured within the same session, making the whole battery less cost-efficient. We therefore decided to focus on individual differences along the susceptibility to the illusion in the expanse of reduced sensitivity to small differences in JNDs.

A different possible source for the lower reliability of the JND scores is related to the way they were measured in the current design. Unlike most previous studies in which JNDs for size differences are presented as simple lines or objects with no surrounding context, JNDs in the current study were measured when the objects were embedded in an illusory context. Wang, Irwin, and Hautus (1998) compared JNDs for line size between nonillusory and illusory conditions (lines embedded in the Müller–Lyer illusion). Their results indicated that the JNDs in the illusory context condition were higher than in the nonillusory condition. We note, however, that Morgan, Hole, and Glennerster (1990) also measured JNDs when the stimuli were presented in illusory and nonillusory contexts and suggested that the illusion does not affect the JNDs. Yet, the two studies used a very small participant sample, which makes it difficult to draw a firm conclusion about the reliability of JNDs measured within the illusory context. Therefore, further experimentation is required in order to test the effect of illusory context on the reliability of JNDs.

Importantly, however, the current results strongly suggest that individual differences along the JNDs could account, at least in part, for individual differences along the susceptibility to illusions. In both experiments, we found positive relationships between the resolution for size difference (JND) and the illusion magnitude, both for the Ponzo and for the Ebbinghaus illusions. This relationship implies that individuals with a lower sensitivity to size differences between the stimuli are prone to larger illusory effects. This pattern of results was replicated in both experiments, despite the relatively lower reliability values of JNDs. This finding implies that the relationship between susceptibility to the illusion and the sensitivity to size differences could be potentially even higher than our findings show. Morgan et al. (1990) used the method of constant stimuli to measure the effect of the Müller–Lyer illusion on JNDs. Their results indicated that, on average, the magnitude of the JNDs was not affected by the illusory display. Although these results could seem contradictory to the current findings, it is important to note that the Morgan et al. (1990) study and our study focused on different aspects of performance. While Morgan et al. (1990) focused on whether JNDs would increase or decrease within the context of the illusion, we focused on the relation between the susceptibilities to the illusions and the JNDs of different individuals. Our results show that individuals with larger (average) JNDs would tend to be more susceptible to the Ponzo and the Ebbinghaus illusions.

The pattern of results for the height–width illusion was somewhat different from the pattern of results obtained for the Ponzo and Ebbinghaus illusions. In particular, there was a negative relationship between the illusion's magnitude and the resolution for size difference, but only in Experiment 2. This negative relationship was unpredicted and might seem counterintuitive at first sight because it suggests that individuals with higher sensitivity (lower JNDs) are more susceptible to the illusion. We can therefore only speculate that this finding could be accounted for by the fact that in the current paradigm, participants were required to compare the sizes of two objects (i.e., the width of two rectangles) as a measure of sensitivity to size differences of the height–width illusion. However, at the same time, the focus in this illusion is on the mechanism that underlies holistic processing *within* the shape of each rectangle (Ganel & Goodale, 2003). Therefore, there is an apparent contradiction between the source of the illusion and the specific paradigm in which it was measured. In particular, the resolution for size differences stems from comparing width *between* objects, while the source of the illusory effect is the holistic perception of shape, which occurs *within* each of the rectangles. In a related study (Wilford & Wells, 2010) explored the hypothesis that holistic processing can reduce the ability to identify changes between stimuli (faces vs. houses). They found that holistic processing reduces identification performance but also improves the ability to detect the occurrence of change (see also Mathis & Kahan, 2014; Poljac, de-Wit, & Wagemans, 2012). Hence, our results could be interpreted in line with the approach that holistic processing is associated with improved change detection between stimuli.

Coren et al. (1976) conducted a pioneer study that suggested that illusions could be categorized into two different groups that share a common mechanism. According to their logic, illusions can be classified into two different groups, each sharing a common perceptual mechanism. Illusions involving distortion on linear extent were classified as Group 1 (Baldwin, horizontal–vertical, Oppel–Kundt, Müller–Lyer, and Sander parallelogram), while illusions of area, shape, and direction were classified as Group 2 (Wundt, Zoellner, Delboeuf, Ebbinghaus, Ponzo, and divided line). More recent studies have tried to identify the relationships between different illusions, beyond classification to different groups. A common finding in all studies is a low yet significant correlation between the susceptibility to the Ponzo and Ebbinghaus illusions, which implies that the illusions’ underlying mechanisms are partly shared. The results of Experiment 1 replicate this relation by showing a small yet reliable association between the susceptibility to the two illusions (Axelrod et al., 2017; Coren & Porac, 1987; Cretenoud et al., 2019; Cretenoud, Francis et al., 2020; Cretenoud, Grzeczkowski et al., 2020; Grzeczkowski et al., 2017). In a similar manner, there was a low yet significant correlation between the susceptibility to the height–width and the Ponzo illusions, indicating that individuals with higher susceptibility to the Ponzo illusion also tend to show a higher susceptibility to the height–width illusion. We note, however, that the results of Experiment 2 did not fully replicate these patterns of correlations between the illusions’ magnitudes. In particular, although there was a positive correlation between the magnitudes of the Ponzo and Ebbinghaus illusions, this correlation was not significant. We believe that the decrease in the reliability of the Ponzo illusion in Experiment 2 could have limited statistical power, which in turn has decreased the reliability of the correlations found between illusions.

Another possible source for the decrease in the reliability of the Ponzo illusion in Experiment 2, as well as for the overall smaller reliability values of the JNDs in both experiments, could be related to the application of an online study design. Although online studies, based on services such as Prolific or Amazon Mechanical Turk, have shown to be effective in many fields of psychology, these studies are generally less controlled in many aspects compared to lab studies. It is therefore entirely possible that some of the participants would be less engaged in the tasks or even completely miss some trials through inattention or hitting the wrong button. Although there is no ideal solution to control for such events, we did try to attenuate their effect on the quality of the data in our analyses of the results. In particular, GOF values of the psychometric curve to each participant's performance were computed in order to ensure data quality for each of the illusions in each session. Given that lapse of attention or random responses would largely decrease GOF values, we applied a strict criterion of GOFs larger than 0.7 for each participant in each of the illusions. In addition, to reduce the possibility of overall less engaged subjects, only participants with GOFs larger than 0.4 in each of the illusions were invited to participate in the second session. The specific criteria and number of participants for which data were excluded in each experiment are described in the Method sections.

Other sources of noise in online studies may compromise data reliability. Such sources may include between-subject variabilities in viewing angle and viewing distance that may limit our tool's sensitivity to detect individual differences in visual resolution to fine changes in size (JNDs). But data reliability can be also compromised by the specific psychophysical method used. For example, although the method of constant stimuli, used here, is a widespread tool for measuring perceptual biases, it is not without its limitations. One such limitation is that subjects can potentially monitor their own biases by noticing the greater frequency of “larger” responses to one of the alternatives and can reduce their biases by “frequency matching.” This could be a factor leading to correlations over subjects. We are aware that there are alternative methods to the method of constant stimuli that could potentially avoid frequency matching and were used for illusions such as the Ebbinghaus and Müller–Lyer in authism (Manning, Morgan, Allen, & Pellicano, 2017). We hope that in the long run, future studies and batteries would extend the present findings by using converging psychophysical measures.

In conclusion, the present study describes a new, online tool to measure susceptibility to visual illusions of size—the BTPI. We have established the reliability of the tool and its sensitivity to tap individual differences along the magnitude of the three illusions (the Ponzo, Ebbinghaus, and height–width illusions), as well as along the perceptual resolution to detect size differences between stimuli embedded within the illusions. In addition, we also describe and discuss novel findings on the relationship between the susceptibility and the resolution to size, as well as the relationship between the different illusions. The computerized tasks and analysis codes are available online for use by researchers in the field. It is our hope that the new tool would be effectively used to study individual and group differences to tap the mechanisms that mediate visual perception of size.
